# Combination of PD98059 and TGF-β1 Efficiently Differentiates Human Urine-Derived Stem Cells into Smooth Muscle Cells

**DOI:** 10.3390/ijms221910532

**Published:** 2021-09-29

**Authors:** Yongha Hwang, Seon-Heui Cha, Donghee Kim, Hee-Sook Jun

**Affiliations:** 1 College of Pharmacy and Gachon Institute of Pharmaceutical Science, Gachon University, Incheon 21999, Korea; sicrios912@naver.com (Y.H.); dh2388@daum.net (D.K.); 2 Department of Marine Bio and Medical Sciences, Hanseo University, Seonsan-si 31962, Korea; sunnycha@hanseo.ac.kr; 3 Lee Gil Ya Cancer and Diabetes Institute, Gachon University, Incheon 21936, Korea; 4 Gachon Medical Research Institute, Gil Hospital, Incheon 21999, Korea

**Keywords:** urine-derived stem cell, diabetes, differentiation of insulin producing cell, pluripotent adult stem cells

## Abstract

Pluripotent adult stem cells have potential applications in cell therapy and tissue engineering. Urine-derived stem cells (UDSCs) differentiate into various cell types. Here, we attempted to differentiate human UDSCs (hUDSCs) into smooth muscle cells (SMCs) using transforming growth factor-beta 1 (TGF-β1) and/or PD98059, an extracellular signal-regulated kinase (ERK) inhibitor. Both quantitative polymerase chain reaction (qPCR) and Western blot analysis showed that the expression of messenger ribonucleic acid (mRNA) and proteins for alpha-smooth muscle actin (α-SMA), calponin (CNN1), and smooth muscle myosin heavy chain (SM-MHC), which are specific markers for SMCs, increased on day 9 after differentiation and again on day 14. The differentiated cells from human UDSCs (hUDSCs) with a combination of TGF-β1 and PD98059 showed the highest expression of SMC marker proteins. Immunocytochemical staining performed to assess the molecular expression revealed CNN and α-SMA colocalizing in the cytoplasm. The cells that differentiated from hUDSCs with a combination of TGF-β1 and PD98059 showed the strongest expression for CNN1, α-SMA, and SM-MHC. Functional testing of the differentiated cells revealed a stronger contractile capacity for the cells differentiated with a combination of PD98059 and TGF-β1 than those differentiated with a single factor. These results suggest the combination of PD98059 and TGF-β1 to be a more effective differentiation method and that differentiated SMCs could be used for restoring the functions of the sphincter muscle or bladder.

## 1. Introduction

Urinary incontinence (UI) is one of the most common diseases in the elderly, especially women, often triggering shame, anxiety, and depression [1]. UI results from the insufficient strength of the bladder and pelvic floor muscles to resist abdominal compression during physical activity. The reduced strength could be attributed to complications associated with senescence, obesity, and diabetes mellitus [2]. Treatment methods for patients with UI include exercise, drug administration, bulking agent injection, and surgical operation [3]. While injecting bulking agents into the urethral tissue requires repeated treatments, the urethral sling surgical method is relatively simple and effective, but leakage may recur after a few years [4]. The smooth muscle cells (SMCs) within the urethra play a key role in determining function of incontinence [5]. To date, there is no cure to restore urethral SMC function. Therefore, UI has been treated, seemingly successfully, in animal models and human patients, using several populations of stem cells [6]. In addition, Nickel et al. [7] suggest that combining the traditional treatment for lower urinary tract symptoms with new methods would have valuable therapeutic effects.

Mesenchymal stem cells (MSCs) obtained from the adipose tissue, bone-marrow, and urine [6,8,9,10] can differentiate into various cells, such as adipocyte, osteocyte, chondrocyte, and myocyte [9,11,12]. Human urine-derived stem cells (hUDSCs) have been reported to possess stem cell-like properties such as proliferative ability, the presence of stem cell surface markers, and multi-potency [6,13,14]. In addition, hUDSCs have several advantages, including an easy and inexpensive isolation procedure. Since the origin of hUDSCs is presumed to be the kidney or bladder [15], they are highly valuable for use as stem cells in the treatment of diseases in these two organs. In particular, the use of hUDSCs is advantageous in cell therapy and tissue engineering applications in bladder tissue repair [8,16].

Transforming growth factor beta 1 (TGF-β1) is known to activate the SMAD signaling pathway using the TGF-β receptor [17,18,19], inducing SMC-related transcription of the genes *ACTA2*, calponin (CNN1), *MYH* encoding alpha-smooth muscle actin (alpha-SMA), and smooth muscle myosin heavy chain (SM-MHC) [20,21]. PD98059, a mitogen-activated protein kinase or extracellular signal-regulated kinase (MAPK/ERK) pathway inhibitor, has been reported to induce SMC differentiation [22]. Previous studies have reported a combination of TGF-β1 and bone morphogenic protein 4 (BMP4), and TGF-β1 and ascorbic acid, respectively, to mediate the differentiation of both adipose tissue-derived and bone marrow-derived stem cells into SMCs [23]. The possibility of the differentiation of hUDSCs into functional SMCs has also been reported [13]; however, the differentiation of hUDSCs into SMCs has not yet been fully elucidated. Therefore, we investigated whether hUDSCs differentiate into SMCs by TGF-β1 or PD98059, and whether the combination of TGF-β1 and PD98059 can enhance the contractile SMC differentiation potential.

## 2. Results

### 2.1. Morphological Changes of the Differentiated SMCs from hUDSCs

**Figure 1:**
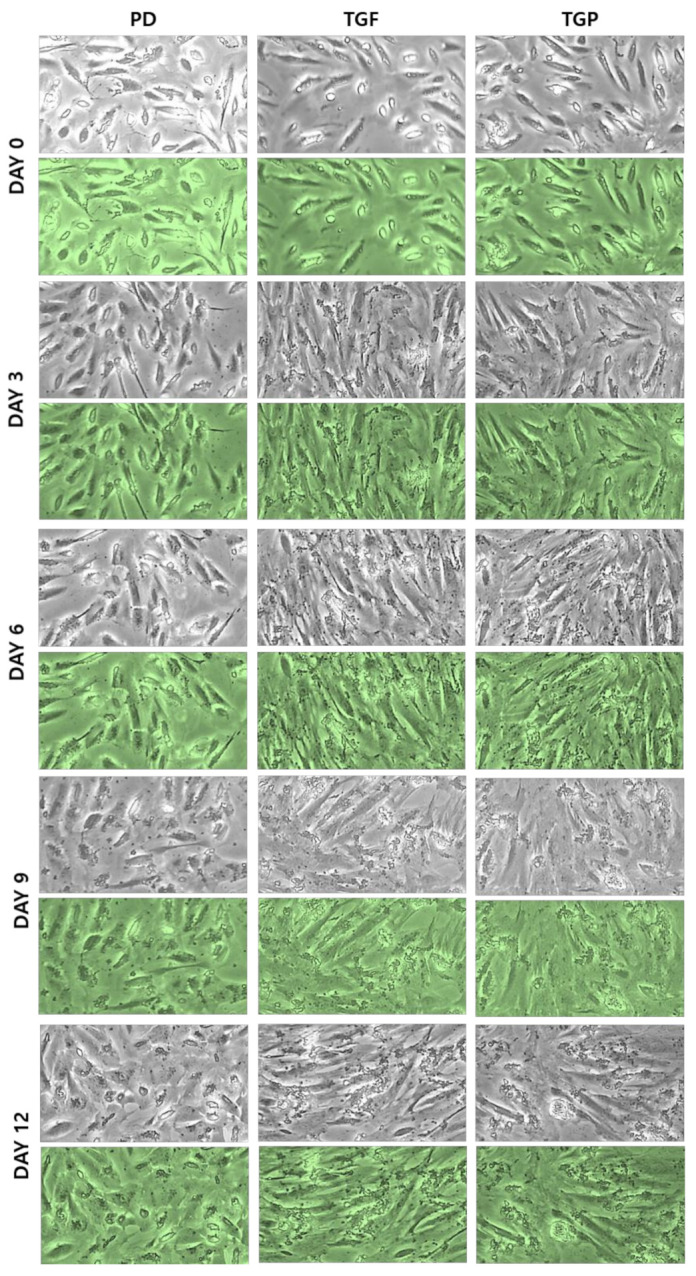
Morphological changes during differentiation of hUDSCs to SMCs using the indicated factors. Morphological changes observed on days 0, 3, 6, 9, and 12 during differentiation of hUDSCs into SMCs. (Magnification, 40×). PD: PD98059, TGF: TGF-β1, TGP: TGF-β1 and PD98059.

It has been reported that MSCs can be differentiated into SMCs by TGF-β1 treatment [24], a cytokine that regulates cell growth, proliferation, and differentiation. The TGF-β-SMAD signaling pathway plays an important role in the induction of differentiation into SMCs. In addition, PD98059 induces stem cell differentiation into SMCs by blocking the ERK signaling pathway [22]. Therefore, we investigated whether these two chemicals, individually or in combination, induced differentiation of the hUDSCs into SMCs. Examining the morphology of the cells during differentiation, a spindle shaped differentiating SMC (a typical SMC phenotype) was observed in all groups. However, the SMC phenotype was clearest in differentiated SMCs with TGF-β1 and a combination of PD98059 and TGF-β1, suggesting that TGF-β1 induced the differentiation of hUDSCs into SMCs (Figure 1).

### 2.2. The Differentiated SMCs from hUDSCs Expressed SMC Marker Proteins, with the Highest Expression in Differentiated Cells with Combination of TGF-β1 and PD98059

**Figure 2:**
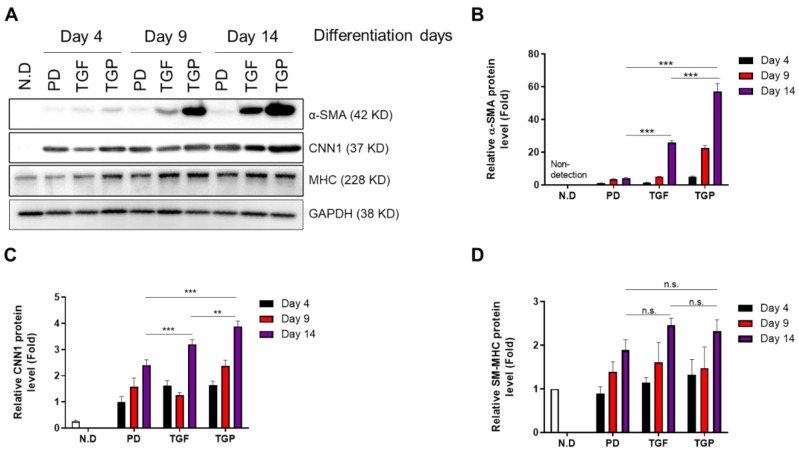
The expression of SMC-specific marker proteins in SMCs differentiated from hUDSCs at day 4, 9, and 14 of differentiation. The expression of α-SMA, CNN1, and SM-MHC was analyzed by Western blot. (**A**) The representative blots are shown. (**B**–**D**) Densitometry quantification of the relative expression of proteins was performed using the ImageJ software. Data are presented as mean ± SEM from three independent experiments; n.s.: no significance, ** *p* < 0.01, *** *p* < 0.005.

To validate whether hUDSCs differentiated into SMCs, the expression of smooth muscle protein markers, including α-SMA, CNN1, and SM-MHC, was determined. The results revealed a time-dependent increase in the expression of these proteins. The α-SMA protein expression was significantly increased at days 9 and 14 in cells differentiated by TGF-β1, whereas PD98059 treatment did not induce α-SMA protein expression. Interestingly, a prominent increase in the α-SMA protein expression was noted when the cells were differentiated using a PD98059–TGF-β1 treatment (Figure 2A,B). The CNN1 protein expression increased from day 4 after differentiation in all groups, but most efficiently in cells differentiated with the PD98059–TGF-β1 combination (Figure 2A,C). An increase in the SM-MHC protein expression was noted from day 9 after differentiation, which further increased at day 14 in cells differentiated with TGF-β1. The SM-MHC protein expression level was similar in cells differentiated using both the PD98059–TGF-β1 combination and TGF-β1 alone (Figure 2A,D).

**Figure 3:**
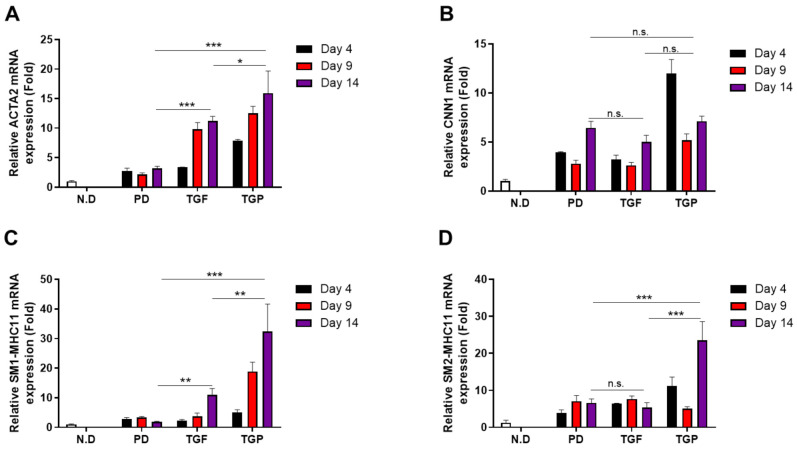
The expression of SMC-specific maker mRNA in SMCs differentiated from hUDSCs at day 4, 9, and 14 of differentiation. The mRNA expression of (**A**) ACTA2 (alpha-smooth muscle actin), (**B**) CNN1 (calponin), (**C**) SM1-MHC11 (smooth muscle 1 myosin heavy chain), and (**D**) SM2-MHC11 (smooth muscle 2 myosin heavy chain) was analyzed by qRT-PCR. The expression of cyclophilin B was used as an internal control: PD; PD98059, TGF; TGF-β1, TGP; PD98059, and TGF-β1. Data are presented as mean ± SEM from three independent experiments. n.s.: no significance, * *p* < 0.05, ** *p* < 0.01, *** *p* < 0.005.

The expression of SMC marker proteins increased during the differentiation of hUDSCs into SMCs. Therefore, we examined mRNA expression of *ACTA2* and *CNN1* during differentiation. The mRNA expression of *ACTA2* and *CNN1* increased from day 4 after differentiation, particularly in cells differentiated with TGF-β1 and a combination of PD98059 and TGF-β1. The cells differentiated with a combination of PD98059 and TGF-β1 showed the highest expression (Figure 3A,B). In addition, the mRNA expression of SM1-MHC11 and SM2-MHC11, known to be expressed in fully differentiated states, also showed similar patterns and combination of PD98059, with TGF-β1 inducing the highest expression (Figure 3D).

### 2.3. The Differentiated SMCs from hUDSCs Decreased the Expression of CD90, Mesenchymal Stem Cell Antigen, with the Highest Reduction in Differentiated Cells with a Combination of TGF-β1 and PD98059

**Figure 4:**
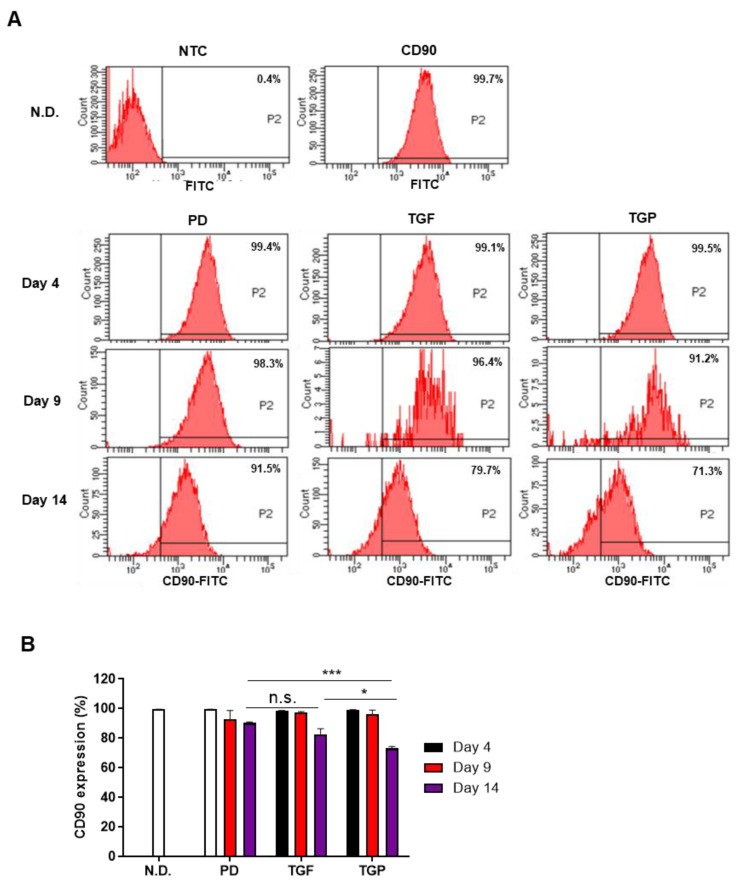
The expression of CD90 in SMCs differentiated from hUDSCs at days 4, 9, and 14 of differentiation. The expression of CD90 was analyzed by flow cytometry. (**A**) The values in the representative flow cytometry histograms indicate the percentages of CD90-expressing cells. (**B**) The percentage of CD90-expressing cells was analyzed from three independent experiments. Data are presented as mean ± SEM from three independent experiments. n.s.: no significance, * *p* < 0.05, *** *p* < 0.005. N.D.; undifferentiated, PD; PD98059, TGF; TGF-β1, TGP; PD98059 and TGF-β1.

To verify whether the expression of MSC marker decreased as hUDSCs differentiated into SMCs, the expression of CD90 was analyzed by flow cytometry. The CD90 expression did not change significantly until day 9 of differentiation; however, it decreased significantly under all conditions on day 14 of differentiation compared to that in the undifferentiated cells or the cells differentiated with PD98059 alone for 4 days (Figure 4A,B). In particular, the CD90 expression at day 14 of differentiation was the lowest when the cells were differentiated using the PD98059–TGF-β1 treatment, which was significantly reduced compared to that in the PD98059 or TGF-β1 alone treatments (Figure 4A,B). These results suggest that the differentiation of hUDSCs into SMCs using the combination of PD98059 and TGF-β1 was more effective in reducing the characteristics of MSCs.

### 2.4. Immunocytochemical Analysis of SMC Markers in the Differentiated SMCs from hUDSCs

**Figure 5:**
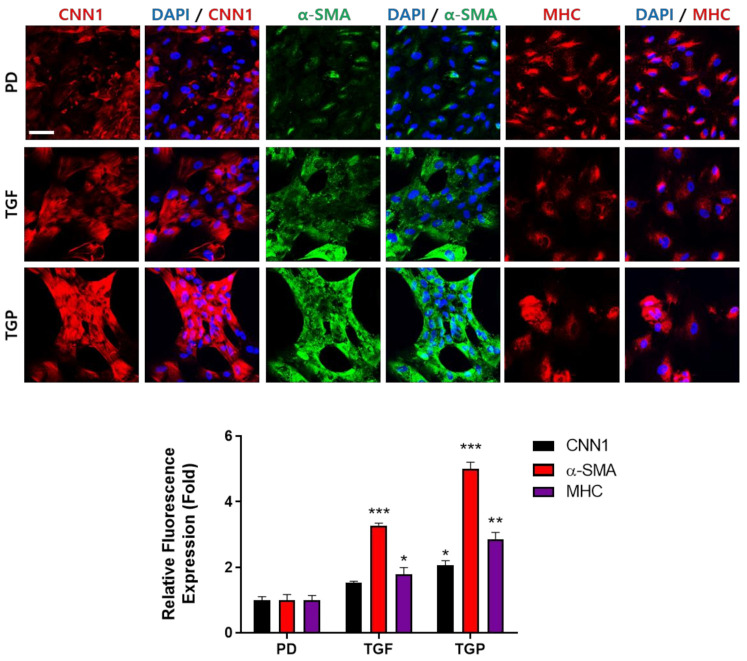
Immunocytochemical analysis of α-SMA, CNN1, and SM-MHC protein expression in differentiated SMCs: PD; PD98059, TGF; TGF-β1, TGP; PD98059 and TGF-β1. Scale bar indicates 50 μm: * *p* < 0.05, ** *p* < 0.01, *** *p* < 0.005, * vs. PD.

Both mRNA and protein expression of α-SMA, CNN1, and SM-MHC were upregulated in a time-dependent manner. The expression level was highest when differentiation was induced by a combination PD98059 and TGF-β1. Therefore, we confirmed the expression of these molecules by immunocytochemical staining and the intensity of three independent experiments was quantified. The mean intensity value of each experiment was acquired by measuring the intensity of at least 100 cells. The CNN1 and α-SMA expression was detected in the cytoplasm. Cells differentiated with the PD98059 and TGF-β1 combination were present in bundles and showed the strongest expression for CNN1, alpha-SMA, and SM-MHC. The expression of α-SMA was minimal in the cells differentiated with PD98059 (Figure 5).

### 2.5. The Differentiated SMCs with a Combination of PD98059 and TGF-β1 Showed the Strongest Contracting Capacity

**Figure 6:**
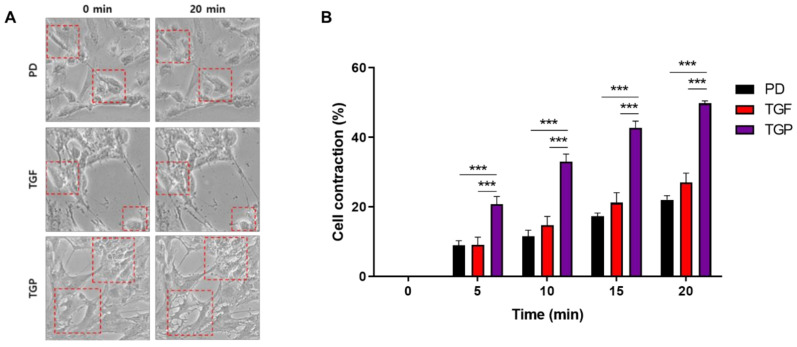
Contractile strength of the differentiated SMCs from hUDSCs. (**A**) Morphology of contractile SMCs. The cells were treated with carbachol to induce contraction, and images were obtained at 0 and 20 min after induction. (**B**) Percentage of carbachol-induced contracting cells. Data are presented as mean ± SEM, *n* = 6: PD; PD98059, TGF; TGF-β1, TGP; PD98059 and TGF-β1. *** *p* < 0.005 vs. PD or TGF group.

Our results showed that induction of cell differentiation by a combination of PD98059 and TGF-β1 is the most efficient marker for smooth muscle expression. The most important function of the smooth muscle when fully matured is a contractile one. To functionally characterize the differentiated cells, a contraction assay was performed using carbachol, a substance specific for smooth muscle contraction. The contraction assay revealed that cells differentiated with PD98059 or TGF-β1 alone exhibit a weak contractile function. On the other hand, the cells differentiated with a combination of PD98059 and TGF-β1 showed an almost two-fold higher contraction ability compared to cells differentiated with a single inducing factor (Figure 6, Supplementary Figure S1). The results thus suggest that use of the PD98059–TGF-β1 combination is more effective in the differentiation of hUDSCs to functional SMCs.

## 3. Discussion

UI is a common problem in older men and women, significantly impacting the quality of life of over 200 million people worldwide [25]. The function of the bladder is to store urine, while that of SMCs is to maintain the urine storage function of the bladder by maintaining proper urethral pressure [26]. However, when there is a problem with the function of the SMCs, urine leakage occurs, resulting in the development of UI disease.

UI is caused by wounds or nerve problems in the urethral sphincter or bladder and can be treated with exercise, drugs, bulking-agent injection, or surgery [2,3,5]. The most fundamental cause of UI is the weakness or lack of SMCs. Most of the treatment strategies are intended to prevent the leakage of urine by inserting a supplement into the urethra, which replaces the function of the weakened SMCs. However, current UI therapies have limitations, including repeated procedures, short-lived therapeutic effects, and a lack of effect on the restoration of bladder or urinary SMCs. The most frequent complications from bulking-agent-injection therapy are dysuria, hematuria, or UI due to other causes [27]. Therefore, the development of alternative therapeutic strategies is required.

Stem cells have attracted attention in the field of regenerative medicine due to their ability to proliferate, self-renew, and differentiate into various cell types. Both undifferentiated and differentiated stem cells have been used to treat UI [28]. The different stem cell sources that have been already used include bone marrow, human cord blood, amniotic fluid, and muscle [29]. Injection of muscle-derived stem cells into the urethra sphincter muscle improved UI-related outcomes [30]. It was also reported that differentiated smooth muscle precursor cells from pluripotent stem cells restored the urethral function in the UI animal models and showed higher leak point pressures (LPP) than the control group [31]. Therefore, different stem cells, such as embryonic, induced-pluripotent, and mesenchymal stem cells, differentiating into SMCs, can be used to restore the function of the sphincter muscle and bladder.

hUDSCs, originating from the kidney or bladder, have several advantages. They can be obtained non-invasively and isolated using low-cost procedures. hUDSCs have the potential to differentiate efficiently into endothelial, urothelial, and SMCs [32]. Therefore, hUDSCs can be used as a good source for stem cells in urological tissue regenerative therapy [6,33]. The use of hUDSCs also holds promise in generating patient-specific SMCs for personalized medicine.

TGF-β has various cellular functions, such as cell growth, migration, apoptosis, and differentiation. It also plays an important role in the differentiation of SMCs [34]. Many studies have reported TGF-β1 to induce SMC differentiation [35,36,37]. TGF-β1 activates the TGFβ receptor kinase and subsequently SMAD2 and SMAD3, contributing to the induction of the expression of SMC-specific markers [37,38,39]. In our study, the cells differentiated from hUDSCs by TGF-β1 expressed α-SMA as an early differentiation marker, and CNN1 and SM-MHC as late differentiation markers in urinary muscles. The high expression of CD90, a representative MSC marker, reflects the undifferentiated state of MSCs. The CD90 level decreased during adipogenic differentiation in vitro of UCB-MSCs [38], and a reduction of CD90 expression using RNA interference enhanced the osteogenic and adipogenic differentiation of MSCs in vitro [39].

PD98059, an inhibitor of the MAPK/ERK pathway, is also known to induce SMC differentiation [22]. Myocardin is a co-activator that binds to the serum response factor (SRF) to promote the expression of SMC markers in cardiac or smooth muscles through CArG elements [40,41]. Myocardin is regulated by the phosphoinositide 3-kinases/protein kinase B (PI3K/AKT) and MAPK/ERK pathways [42] and competitively binds to SRF with phosphorylated ETS like-1 (Elk-1). Phosphorylated Elk-1 reduces the expression of SMC markers by replacing the SRF bound myocardin. Thus, the binding of myocardin to SRF can be improved by blocking the phosphorylation of Elk-1 using a MAPK/ERK inhibitor. MAPK/ERK inhibitors induced the expression of SM22α, one of the SMC markers, in bone marrow stem cells but did not induce the expression of other markers such as SM-MHC and alpha-SMA [22]. We reported increased expression of SMC markers, such as CNN1, α-SMA, and SM-MHC, during differentiation of hUDSCs with PD98059. However, the expression of SMC markers was weak compared to the cells differentiated with TGF-β1.

In our study, we used a combination of TGF-β1 and PD98059 to develop a more efficient method for the differentiation of hUDSCs to SMCs. The differentiated SMCs showed the highest expression for α-SMA, CNN1, and SM-MHC and were synergistically induced compared to the cells differentiated with PD98059 or TGF-β1 alone. Similarly, the function of the differentiated SMCs with a combination of PD98059 or TGF-β1, evidenced by carbachol-induced contractile ability, was also the strongest, showing a synergistic effect compared to when differentiated with a single inducing factor. Therefore, the combination of PD98059 and TGF-β1 is considered to be more effective in the differentiation of hUDSCs into SMCs.

In this study, we found that hUDSCs isolated from human urine can be differentiated into SMCs using PD98059 or TGF-β1. When hUDSCs were differentiated with a combination of PD98059 and TGF-β1, the differentiated SMCs showed a much higher expression of SMC marker proteins and stronger contraction ability. Although animal studies are needed, SMCs differentiated from hUDSCs with the PD98059–TGF-β1combination could be a potential cell source for regeneration therapy to treat UI.

## 4. Materials and Methods

### 4.1. Ethical Approval

The use of hUDSCs from healthy donors (11 males, aged 23–51 years) in this study was reviewed and approved by the Institutional Review Board of the Gil Medical Hospital (1044396-201809-HR-180-01).

### 4.2. Maintenance of hUDSCs

Human UDSCs were isolated in a previous study [43]. The isolated cells were cultured in a medium containing 1:1 mixture of Dulbecco’s modified eagle medium: Nutrient mixture F-12 (DMEM/F12; Thermo Fisher Scientific, Waltham, MA, USA), keratinocyte serum-free medium (KSFM; Thermo Fisher Scientific), 5% fetal bovine serum (FBS; Thermo Fisher Scientific), and 1% penicillin/streptomycin (Welgene, Deagu, Korea). The hUDSCs were maintained in a humidified incubator at 37 °C and 5% carbon dioxide (CO_2_). The culture media was changed every two days. Once the cells attained 70–80% confluency, they were deemed fit for use. The cells were next seeded (1 × 10^4^ cells/well) in 24-well plate (Falcon, Corning, NY, USA) and incubated at 37 °C, 5% CO_2_ in an air humidified incubator (Heraeus HERACell 150, Thermo Fisher Scientific). The culture medium was changed every two days until the cells formed colonies. Each colony was then transferred to individual wells of a 24-well plate and cultured. The cells were split when they reached 70–80% confluency. All hUDSCs phenotypes were confirmed as shown in our previous study [44].

### 4.3. SMCs Differentiation

The hUDSCs were seeded (4.0 × 10^5^ cells/well) onto a laminin (120 μg/mL) coated 6-well plate (BD Falcon, Bedford, MA, USA) and incubated until the cell confluency reached 100%. The cells were then incubated with the SMC differentiation medium. The seeded cells were incubated with 10% FBS and 1% penicillin/streptomycin, containing high-DMEM (Welgene, Deagu, Korea). The cells were also supplemented with 10 µM PD98059 (Calbiochem, San Diego, CA, USA) and/or 10 ng/mL TGF-β1 (Peprotech, Rocky Hill, NJ, USA). The culture medium was changed every 3 days. After 14 days, the cells were harvested and used according to each experimental method.

### 4.4. Quantitative Real-Time Polymerase Chain Reaction (qRT-PCR)

Cells were harvested on the 4, 9, and 14 days of differentiation. Total ribonucleic acid (RNA) was extracted from the cells using RNAiso Plus (Takara Bio, Kusatsu, Japan), according to the manufacturer’s protocol. Complementary deoxyribonucleic acid (cDNA) was synthesized from 2 μg of total RNA using the PrimeScript™ cDNA synthesis kit (Takara Bio), according to the manufacturer’s protocol. The samples of cDNA were then analyzed using the SYBR^®^ Premix Ex Taq™ and ROX plus (Takara Bio) on Bio-Rad cyclers (Bio-Rad, Hercules, CA, USA). Gene expression was normalized to the endogenous housekeeping control gene, cyclophilin. Relative expression was calculated for each gene using the ΔΔCT (where CT is the threshold cycle) method. The primer sequences used are listed in Table 1 [43].

### 4.5. Flow Cytometry

Cells were seeded (2.0 × 10^5^ cells/well) on laminin (120 μg/mL) coated 12 well plates, after which differentiation was induced. Cells were harvested on the 0, 4, 9, and 14 days of differentiation and were suspended in 1% FBS in PBS. Cells were stained with the FITC-conjugated anti-human CD90 (Thy1) antibody (Cat.#. 328107; BioLegend Inc., San Diego, CA, USA) at 4 °C for 30 min in the dark. Cells were washed twice with 1% FSB in PBS and were analyzed using a FACS LSR II equipped with FACSDiva™ Software (Becton Dickinson Biosciences, San Jose, CA, USA).

### 4.6. Western Blotting

Cells harvested on the 4, 9, and 14 days of differentiation were lysed on ice for 20 min with a radioimmunoprecipitation assay (RIPA) lysis buffer containing a protease inhibitor cocktail (GenDEPOT, Harris County, TX, USA). The lysates were centrifuged at 12,000 rpm for 30 min at 4 °C and transferred to a new 1.5 mL tube. The protein concentrations were measured using a bicinchoninic acid (BCA) assay kit (Thermo Fisher Scientific, Waltham, MA, USA). The lysates were separated by sodium dodecyl sulfate–polyacrylamide gel electrophoresis (SDS-PAGE) and transferred to polyvinylidene fluoride (PVDF) membranes (Millipore, Billerica, MA, USA). The membranes were incubated with 5% skim milk at room temperature for 1 h, then incubated overnight at 4 °C with primary antibodies, anti-alpha-SMA (Abcam, Cambridge, UK), anti-CNN1 (Abcam), and anti-SM-MHC (Abcam). After washing extensively, the membranes were incubated with the horseradish peroxidase (HRP)-conjugated secondary antibody (Jackson ImmunoResearch, West Grove, PA, USA) for 2 h. The signal was detected using WESTSAVE (AbFrontier, Korea) and an enhanced chemiluminescence system. ImageJ (https://imagej.nih.gov/ij/,download.html, accessed on 14 September 2021) software was used to quantify the band intensity of the Western blot.

### 4.7. Characterization of SMCs by Immunocytochemistry

Cells were seeded (1.0 × 10^5^ cells/well) on laminin (120 μg/mL) coated cover glasses and differentiation was induced. After 14 days of differentiation, the cells were washed with phosphate-buffered saline (PBS) and fixed using 4% paraformaldehyde in PBS at 4 °C overnight. The fixed cells were then: (1) washed with PBS, (2) blocked with PBS containing 1% bovine serum albumin (BSA) and 0.3% Triton X-100 for 1 h at room temperature, and (3) incubated overnight with anti-alpha-SMA, anti-CNN1, and anti-SM-MHC (Abcam, UK) at 4 °C. Staining of the cells was performed next using a fluorescence-conjugated, fluorescein isothiocyanate (FITC), or Alexa 546, secondary antibody (Life Technologies, Carlsbad, CA, USA) for 2 h at room temperature. The cells were washed twice with PBS and stained with 4′,6-diamidino-2-phenylindole (DAPI) (5 μg/mL) for 10 min. After DAPI staining, the cells were washed twice with PBS, transferred to micro-slide glass (Matsunami Glass, Osaka, Japan), and mounted with VECTASHIELD (Vector Laboratories, Burlingame, CA, USA). A confocal microscope (Zeiss, Jena, Germany) was used to observe the cells. The expression of alpha-SMA, CNN, and SM-MHC was evaluated by quantifying the fluorescence intensity using the ImageJ software.

### 4.8. Contraction Test

Carbachol-induced contractibility assays were performed as described previously [45]. Briefly, after 14 days of differentiation, the cells were washed twice with PBS and incubated with a non-enzyme dissociation buffer (Sigma, St. Louis, MO, USA) for 30 min. The cells were then treated with a 10 μM carbachol (Cayman Chemical, Ann Arbor, MI, USA) solution for 20 min. Images were obtained every 5 min after treatment with carbachol, with the contraction assessed by measuring the cell cross-sections using the ImageJ software.

### 4.9. Statistical Analysis

Statistical analysis was performed using a one-way analysis of variance (ANOVA) with the Bonferroni test. Data are presented as mean ± standard error of the mean (SEM). The observations were considered significant at *p* < 0.05.

## Figures and Tables

**Table 1:** Primer list and sequences for qRT-PCR.

Gene	Accession Number	Primer Name	Primer Sequence (5′-3′)	Aneling Temperature (°C)	Amplification Size (bp)
Actin alpha 2, smooth muscle (ACTA2)	NM_001613.4	ACTA2-F ACTA2-R	ACTGCCTTGGTGTGTGACAA TCAAACCCCAATCCACAGAG	55	120
Calponin 1 (CNN1),	NM_001299.6	CNN1-F CNN1-R	AGGTTAAGAACAAGCTGGCCC GAGGCCGTCCATGAAGTTGT	55	113
Myosin heavy chain 11 isoform SM1 (MYH11 SM1)	NM_002474.2	MYH11-SM1-F MYH11-SM1-R	CAAGAGCAAGCTCAGGCGA CCTCCTCAGAACCATCTGCATT	55	98
Myosin heavy chain 11 isoform SM2 (MYH11 SM2)	NM_022844.2	MYH11-SM2-F MYH11-SM2-R	GATGCACCAGGCGAGGAAA TGAAGTCTGCGTCTCGAGTG	55	120
Cyclophilin B	NM_000942.5	Cyclo-F Cyclo-R	TGCCATCGCCAAGGAGTAG TGCACAGACGGTCACTCAAA	55	57

## Data Availability

The corresponding author will serve the datasets of this study on reasonable request.

## Supplementary Materials

The following are available online at https://www.mdpi.com/article/10.3390/ijms221910532/s1.
