# Supplementary figures and images for: Combination of PD98059 and TGF-β1 Efficiently Differentiates Human Urine-Derived Stem Cells into Smooth Muscle Cells
Source: Int J Mol Sci. 2021 Sep 29;22(19):10532. doi: 10.3390/ijms221910532 (PMC8508912; doi:10.3390/ijms221910532)

Supplementary Video. Contraction test

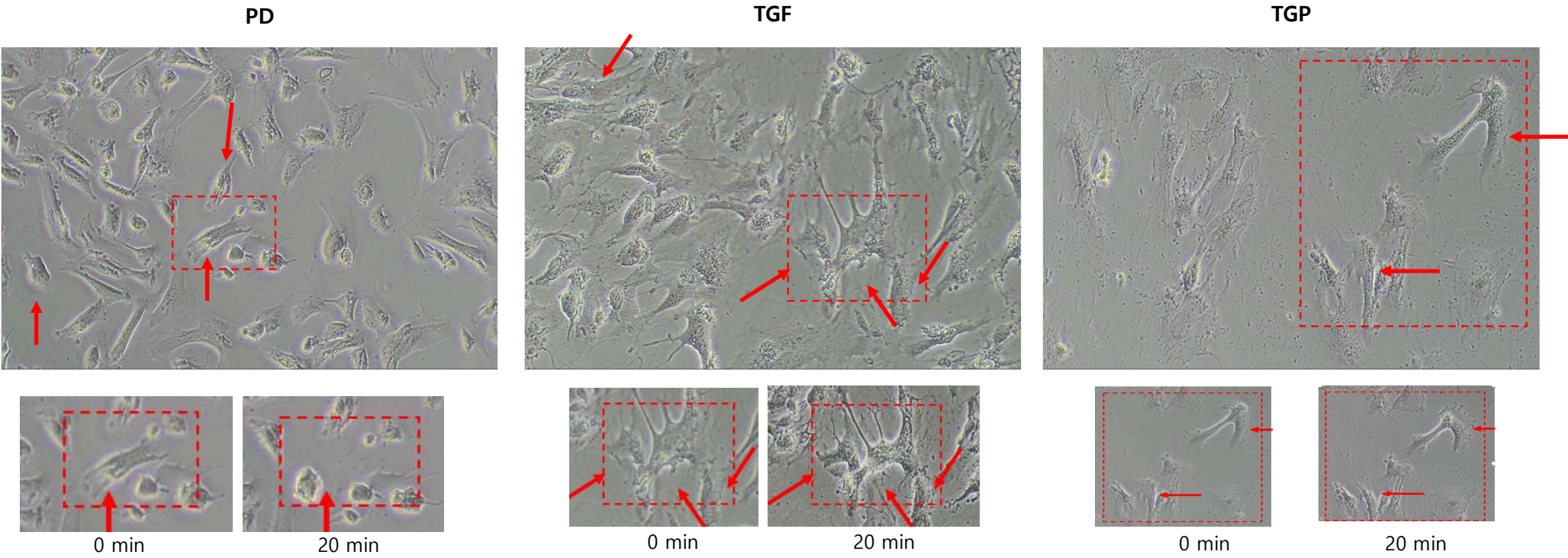

PD

TGF

TGP

0 min

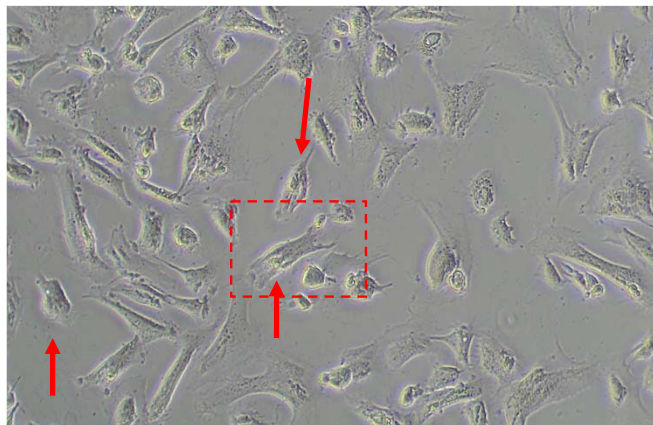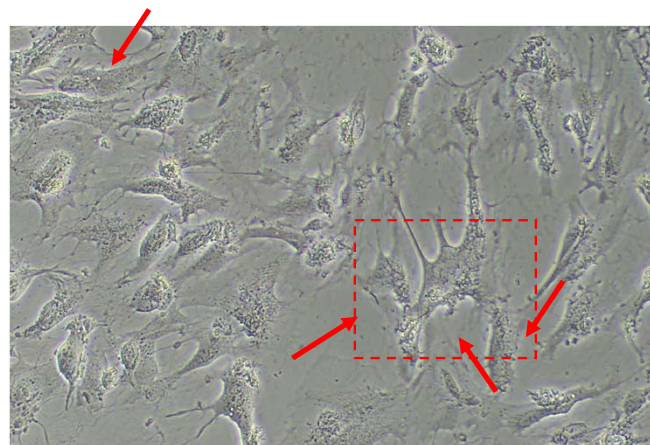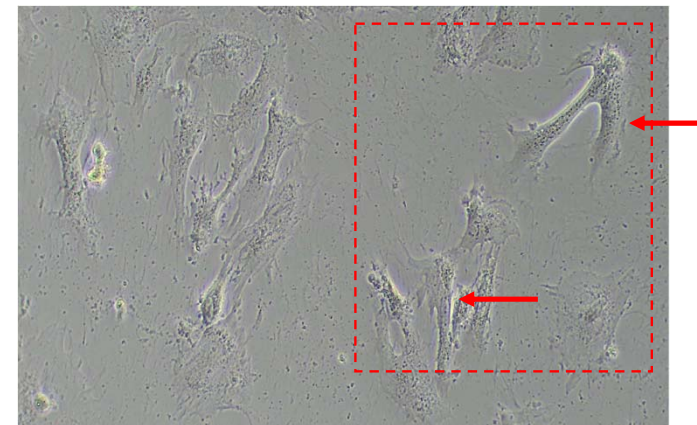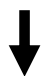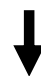

20 min

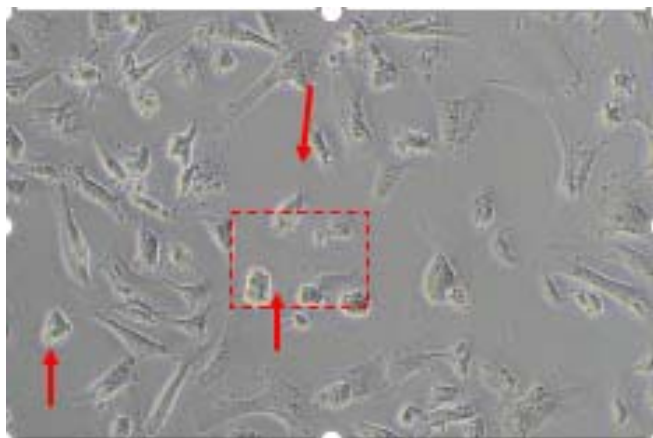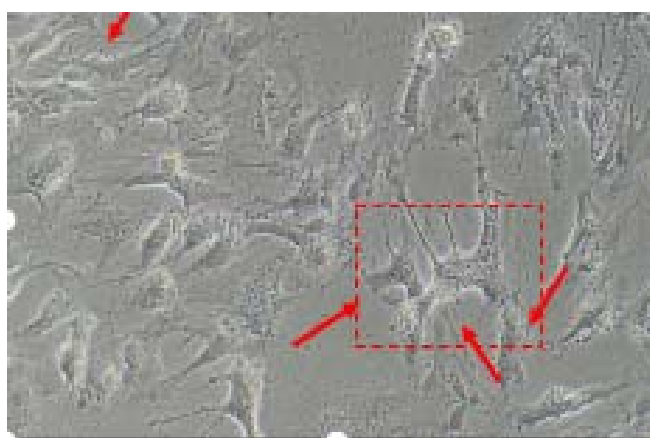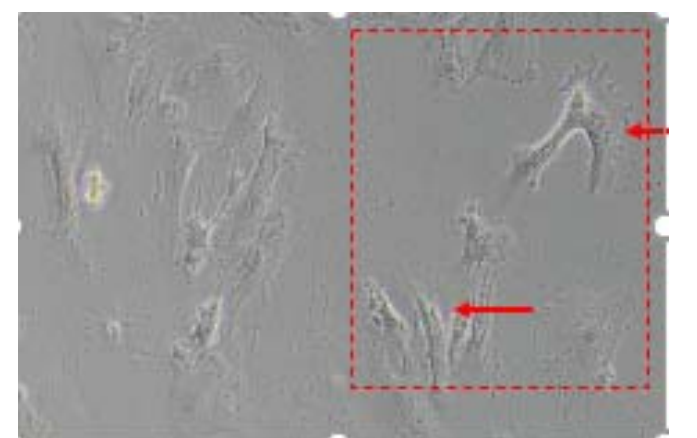

Supplement: Supplementary file 1 [file ijms-22-10532-s001.zip › ijms-1280055-supplementary.pdf]
